# Dual functions of discoidinolysin, a cholesterol-dependent cytolysin with N-terminal discoidin domain produced from *Streptococcus mitis* strain Nm-76

**DOI:** 10.1080/20002297.2022.2105013

**Published:** 2022-08-01

**Authors:** Atsushi Tabata, Airi Matsumoto, Ai Fujimoto, Kazuto Ohkura, Takuya Ikeda, Hiroki Oda, Shuto Yokohata, Miho Kobayashi, Toshifumi Tomoyasu, Ayuko Takao, Hisashi Ohkuni, Hideaki Nagamune

**Affiliations:** a Department of Bioengineering, Division of Bioscience and Bioindustry, Graduate School of Technology, Industrial and Social Sciences, Tokushima University Graduate School, Tokushima, Japan; b Department of Biological Science and Technology, Life System, Institute of Technology and Science, Tokushima University Graduate School, Tokushima, Japan; c Department of Bioengineering, Faculty of Bioscience and Bioindustry, Tokushima University, Tokushima, Japan; d Division of Clinical Pharmacy and Pharmaceutical Sciences, Graduate School of Pharmaceutical Sciences, Suzuka University of Medical Science, Suzuka, Japan; e Department of Oral Microbiology, School of Dental Medicine, Tsurumi University, Kanagawa, Japan; 6 Research Institute, Health Science Research Institute East Japan Co., Ltd., Saitama, Japan

**Keywords:** Discoidinolysin, *Streptococcus mitis*, Mitis group streptococci, cholesterol-dependent cytolysin, discoidin domain, β-hemolysis, cytotoxicity, virulence factor

## Abstract

**Background:**

Some strains of *Streptococcus mitis* exhibit β-hemolysis due to the β-hemolytic activity of cholesterol-dependent cytolysin (CDC). Recently, a gene encoding an atypical lectinolysin-related CDC was found in *S. mitis* strain Nm-76. However, the product of this gene remains uncharacterized. We aimed to characterize this atypical CDC and its molecular functions and contribution to the pathogenicity of *S. mitis* strain Nm-76.

**Methods:**

Phylogenetic analysis of the CDC gene was conducted based on the web-deposited information. The molecular characteristics of CDC were investigated using a gene-deletion mutant strain and recombinant proteins expressed in *Escherichia coli*.

**Results:**

The gene encoding CDC found in Nm-76 and its homolog are distributed among many *S. mitis* strains. This CDC is phylogenetically different from other previously characterized CDCs, such as *S. mitis*-derived human platelet aggregation factor (Sm-hPAF)/lectinolysin and mitilysin. Because this CDC possesses an additional N-terminal domain, including a discoidin motif, it was termed discoidinolysin (DLY). In addition to the preferential lysis of human cells, DLY displayed N-terminal domain-dependent facilitation of human erythrocyte aggregation and intercellular associations between human cells.

**Conclusion:**

DLY functions as a hemolysin/cytolysin and erythrocyte aggregation/intercellular association molecule. This dual-function DLY could be an additional virulence factor in *S. mitis*.

## Introduction

*Streptococcus mitis* is an opportunistic pathogen inhabiting the human oral cavity. Although *S. mitis* is genetically similar to the well-known human pathogen *Streptococcus pneumoniae*, the clinical importance of *S. mitis* has gained lesser focus compared with *S. pneumoniae*. However, recently, clinical reports of disorders caused by *S. mitis* have increased. These reports have described streptococcal toxic shock syndrome [1–3] and the association of *S. mitis* with ectopic disorders, such as endocarditis [4–10]. Therefore, understanding the potential pathogenicity of *S. mitis* is important.

For the strains belonging to the Mitis group streptococci (MGS), several proposals for new species [11–17], reclassification of the traditionally accepted species [18], and phylogenetic analysis [19] have been described in the past decade. For example, *Streptococcus pseudopneumoniae* is a new species in the MGS [20] that shares common genes with *S. pneumoniae* and *S. mitis* [21]. Based on comparative genome analysis, it has been suggested that this species may be a human pathogen [22–24]. Several reports on the pathogenicity of *S. pseudopneumoniae* in humans have been published [25–27]. Genetic and transcriptomic analyses of *S. pneumoniae*-related species among the MGS have been performed [28–30]. Consequently, some *S. mitis* strains that possess genes encoding the homologs of pathogenic factor(s) of *S. pneumoniae* have been recognized [31–34]. These strains may constitute a human-pathogenic subgroup of *S. mitis*.

Cholesterol-dependent cytolysin (CDC) is a pore-forming toxin secreted from both human-pathogenic and human-opportunistic Gram-positive bacteria. A CDC termed intermedilysin (ILY) was discovered in the human-opportunistic species *Streptococcus intermedius* belonging to the Anginosus group streptococci [35]. ILY is important for the pathogenicity of *S. intermedius* in humans [36]. In addition, investigations on the pathogenicity of other opportunistic streptococci that inhabit the oral cavity of humans revealed that a group of *S. mitis* strains displayed obvious β-hemolysis on blood agar [37]. The first reported β-hemolytic factor produced by *S. mitis*, mitilysin (MLY), is a typical CDC composed of four domains with extensive homology to pneumolysin (PLY) [38]. Subsequently, *S. mitis*-derived human platelet aggregation factor (Sm-hPAF), originally identified as a platelet aggregation factor [39], was shown to function as a hemolysin [40]. Sm-hPAF is a CDC with a remarkable structural characteristic of an additional N-terminal domain attached to the four-domain structure of typical CDCs. The name lectinolysin (LLY) was proposed for this CDC based on the results of the functional analysis of the N-terminal additional domain of the Sm-hPAF homolog from *S. mitis* strain SK597 [41]. Genes encoding PLY-like proteins, including the F5_F8_type_C domain, also known as the discoidin domain or C2-like domain – a major domain of many blood coagulation factors [42] – were also found in the genome of *S. pseudopneumoniae* IS7493 [SPPN_02090 (present locus tag is SPPN_RS02030) and SPPN_04220] [29]. In addition, different subfamilies of SPPN_02090 orthologous genes, designated *llyA2*, were found in *S. mitis* strains [29]. However, the expression of the *llyA2* transcription product and its molecular functions have not yet been investigated.

During our investigation of the β-hemolytic subgroup strains of *S. mitis*, we found that the strain Nm-76 possessed a gene encoding an atypical CDC. We observed that this atypical CDC possesses an additional N-terminal domain containing the F5_F8_type_C domain on which other noteworthy characteristics of the CDC depend. Therefore, we designated this atypical CDC as discoidinolysin (DLY). DLY was slightly different from the predicted *llyA2* product and was phylogenetically different from both MLY and Sm-hPAF/LLY. In the present study, we confirmed the expression of *dly* in strain Nm-76 immunochemically and biochemically using immunoblotting and hemolytic activity assays. The distribution of *dly* among the MGS was investigated using the information deposited in the web-based database. Moreover, we constructed a *dly*-deletion mutant and compared its phenotype with that of the Nm-76 wild strain. Several derivative recombinant proteins of DLY were also constructed using an *Escherichia coli* expression system, and the detailed molecular characteristics of DLY derived from *S. mitis* strain Nm-76 were investigated.

## Materials and methods

### Bacterial strains and culture

The strains used in this study were *S. mitis* strain Nm-76 isolated from the oral cavity of a patient with Kawasaki disease, a mutant strain with the gene encoding DLY deleted, and the non-hemolytic *S. mitis* strain NS51^T^ (GTC 495^T^, NCTC 12261^T^). *S. mitis* strain Nm-76 was isolated at Nippon Medical School Hospital (Tokyo, Japan) under contract and was used in accordance with the ethical guidelines provided by the Japanese Society for Bacteriology. *S. mitis* strain NS51^T^ was provided by the Gifu University Center for Conservation of Microbial Genetic Resources (GCMR). These strains were cultivated in brain-heart infusion (BHI) broth (Becton Dickinson and Co., Franklin Lakes, NJ) at 37°C and 5% CO_2_.

### Determining the nucleotide sequence

Genomic DNA from *S. mitis* strain Nm-76 was purified as previously reported [43]. The nucleotide sequence of *dly* was determined by amplicon sequencing using primers 1–4 listed in Table S1. Nucleotide sequencing was performed by BEX Co. Ltd. (Tokyo, Japan). The determined nucleotide sequence was submitted to the DDBJ/eMBL/GenBank database under accession number LC618822.

### Phylogenetic analysis

A BLASTn search of the CDC genes was conducted using the databases *S. mitis* Nucleotide BLAST (https://www.ncbi.nlm.nih.gov/genome/?term=Streptococcus-mitis) and *S. pseudopneumoniae* Nucleotide BLAST (https://www.ncbi.nlm.nih.gov/genome/?term=streptococcus-pseudopneumoniae). For comparison, genes encoding Sm-hPAF and their homologs, including LLY, and genes encoding MLY and PLY were also selected. The gene encoding streptolysin O (SLO), derived from *S. pyogenes* MGAS5005, was selected as the outgroup. The nucleotide sequence information is presented in Table S2. Phylogenetic analysis was performed using MEGA7 [44] with default parameters.

### Molecular modeling

The molecular model of DLY from *S. mitis* strain Nm-76 was constructed based on the structural data of ILY (PDB ID:1S3R) and the lectin domain of LLY (PDB ID:3LE0) using Insight II-Discover with a homology module (Accelrys Inc., San Diego, CA) as previously described [45,46].

### Constructing the gene-deletion mutant

The *dly*-deletion mutant of *S. mitis* strain Nm-76 was constructed using homologous recombination [47]. Briefly, the upstream and downstream fragments of *dly* were amplified using PCR with PrimeSTAR Max DNA polymerase (TaKaRa Bio Inc., Shiga, Japan), primers 5–8 listed in Table S1, and purified genomic DNA as the template. The chloramphenicol acetyltransferase (*cat*) gene cassette was also amplified using PrimeSTAR Max DNA polymerase (TaKaRa Bio Inc.) with primers 9 and 10 (Table S1) and pMX2 [48] as the template. The amplicons were purified using NucleoSpin Gel and PCR Clean-up (TaKaRa Bio Inc.) and fused to generate a single fragment by fusion PCR using PrimeSTAR Max DNA polymerase (TaKaRa Bio Inc.) with primers 5 and 8. The purified fragments were used for the natural transformation of Nm-76 in the presence of a competence-stimulating peptide (EMRRIGSVLLNFFKRR; CSBio Inc., Menlo Park, CA) as described previously [47]. The *dly*-deletion mutant was screened using PCR with GoTaq Green Master Mix (Promega Corp., Madison, WI) and primers 9 and 10 (Table S1). To confirm the sequence of the selected clones, the amplicons prepared using PrimeSTAR GXL DNA polymerase (TaKaRa Bio Inc.) were sequenced by Eurofins Genomics K.K. (Tokyo, Japan) using primers 11–14 (Table S1). DLY production was checked using immunoblotting with murine antiserum (AS) as the primary antibody. AS was generated in our laboratory against the N-terminal domain of DLY (discoidin domain-containing domain, designated as DD) as the antigen under Protocol No. T29-38 approved by the Committee on Animal Experiments of Tokushima University (Tokushima, Japan) [49]. Bacterial growth of the *dly*-deletion mutant was almost the same as that of the wild-type strain Nm-76 in both BHI broth and the co-cultivation medium described below.

### Expression system of recombinant proteins

**Figure 1.**
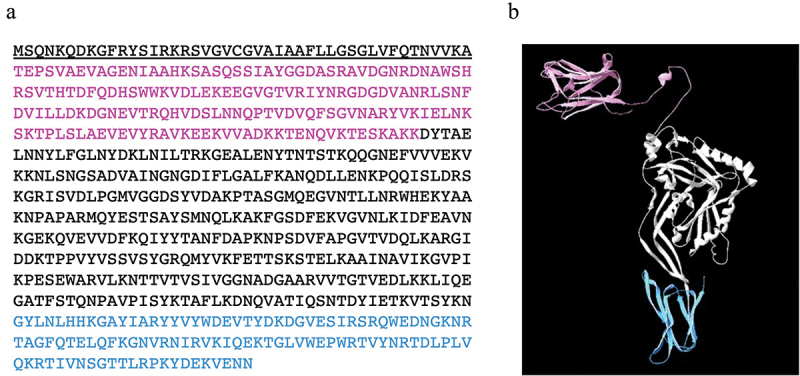
Amino acid sequence (a) and predicted three-dimensional structure (b) of discoidinolysin (DLY), the atypical CDC produced by the S. mitis strain Nm-76. The N-terminal domain, which includes a discoidin domain (DD), and a receptor recognition domain (domain 4) are colored in magenta and light blue, respectively. The amino acid sequence underlined in (a) is the secretion signal sequence.

The recombinant protein expression system was constructed according to a previously reported method [50]. Briefly, to construct recombinant DLY (rDLY), the fragment encoding DLY without a predicted signal sequence consisting of 44 amino acids, as underlined in Figure 1a, was amplified using PrimeSTAR HS DNA polymerase (TaKaRa Bio Inc.) with primers 3 and 4 (Table S1) and genomic DNA purified from *S. mitis* Nm-76 as the template. The amplified fragment was digested by both BamHI and HindIII and purified and cloned into pQE-9 (Qiagen, Hilden, Germany) using DNA Ligation Kit <Mighty Mix> (TaKaRa Bio Inc.). Subsequently, *E. coli* JM109 competent cells were transformed with the vector. The target clones were selected after PCR using GoTaq DNA polymerase (Promega Corp.) with primers 15 and 16 (Table S1), and isolated colonies were used as templates. Sequencing of the purified plasmids from PCR-positive clones was outsourced (BEX Co., Ltd.). The expression systems of the DD and DD-deleted DLY (ΔDD) were also constructed according to the method described above using a primer sets: 3 and 17 and 18 and 4, respectively (Table S1).

The expression systems for recombinant proteins that prevented pore-forming activity (designated as the name of recombinants followed by (ss)) were also constructed. To block the conformational change essential for pore formation, expression systems of prepore-locked recombinants with two-point mutations (G240C and N371C) in domains 2 and 3 were constructed using PCR-based mutation and In-Fusion cloning. Briefly, the insert fragment was prepared using fusion PCR with a high-fidelity DNA polymerase (PrimeSTAR GXL DNA polymerase and PrimeSTAR HS DNA polymerase, TaKaRa Bio Inc.) with primers 19–28 (Table S1). To amplify the vector fragment, PrimeSTAR Max DNA polymerase (TaKaRa Bio Inc.) was used with primers 29 and 30 (Table S1). Ligation of these fragments was conducted using an In-Fusion HD Cloning Kit (TaKaRa Bio Inc.), and *E. coli* DH5αZ1 competent cells were transformed with the ligation product. Clone screening was performed as described above. In addition, using the constructed plasmid, another expression system for the prepore-locked recombinant without DD was also constructed: for the amplification of the insert, PrimeSTAR HS DNA polymerase (TaKaRa Bio Inc.) was used with primers 31 and 32 (Table S1); the PrimeSTAR Max premix (TaKaRa Bio Inc.) with primers 29 and 30 (Table S1) was used to amplify the vector fragment; using these fragments. In-Fusion cloning and subsequent transformation of *E. coli* DH5αZ1 were conducted as described above. Furthermore, an expression system for prepore-locked recombinants with three Ala-substituted mutations (H93A, R120A, R128A, Figure S1) at the fucose-binding residues reported previously [41] was constructed using PCR mutagenesis with PrimeSTAR Max premix (TaKaRa Bio Inc.), primers 33–36 (Table S1), and the constructed vector of the prepore-locked recombinant described above as the template. *E. coli* DH5αZ1 cells were transformed using the PCR product described above, and the sequence of the constructed vector was outsourced to Eurofins Genomics K.K. (Tokyo, Japan).

### Purifying the recombinant proteins

The recombinant proteins were purified using AKTAprime plus (Cytiva, Marlborough, MA, USA) equipped with a HisTrap HP column (Cytiva) [50]. The purity of the recombinant proteins was estimated using standard Coomassie Brilliant Blue staining after Laemmli’s sodium dodecyl sulfate (SDS)-polyacrylamide gel electrophoresis [51]. The concentration of each purified protein was determined using the Bradford method with Protein Assay Dye Reagent (Bio-Rad, Hercules, CA).

### Observing membrane binding and prepore formation of mutant recombinants

The membrane binding and prepore formation activities of the DLY recombinants possessing an intramolecular disulfide bond were observed using immunoblotting against DD of DLY after SDS-agarose gel electrophoresis (SDS-AGE). Each recombinant protein was incubated with PBS-washed human erythrocytes in the presence or absence of 10 mM dithiothreitol (DTT) at 37°C for 1 h. After incubation, the reaction mixture was centrifuged (21,100 × *g*, 4°C, 5 min)  and the pellet was washed once with PBS (21,100 × *g*, 4°C, 5 min). The washed pellet was dissolved in sample buffer for SDS-AGE (50 mM Tris-HCl (pH 6.8), 2% (w/v) SDS, 0.025% (w/v) BPB, and 5% (w/v) glycerol) and applied to a 1.5% (w/v) agarose gel prepared using electrophoresis buffer (25 mM Tris, 1.44% (w/v) glycine, and 0.1% (w/v) SDS). Electrophoresis was performed at 50 V, and the separated proteins were electrically transferred to a polyvinylidene difluoride (PVDF) membrane using standard semi-dry blotting. The blotted membrane was blocked with blocking solution (Tween 20-added Tris-buffered saline (TTBS) containing 1.0% (w/v) skim milk), followed by incubation with the AS described above as the primary antibody and horseradish peroxidase (HRP)-conjugated anti-mouse IgG polyclonal antibody (SeraCare Life Sciences, Inc., Milford, MA, USA) as the secondary antibody. Finally, the membrane was reacted with the substrate solution (Immobilon Western Chemiluminescent HRP Substrate; Merck Millipore) and detected using an LAS-4000 mini EPUV (FUJIFILM, Tokyo, Japan).

### Hemolysis assay

Human erythrocytes were prepared from the blood of healthy volunteers who provided written informed consent in accordance with Protocol No. 15002 approved by the Institutional Ethics Review Board at the Institute of Technology and Science, Tokushima University Graduate School. Sterilized preserved animal blood (horse, rabbit, and sheep blood) was purchased from Nippon Bio-Supply Center (Tokyo, Japan). The supernatant prepared from overnight cultures of the tested strains and purified recombinant proteins were used to assay hemolytic activity. The final concentration of the culture supernatant was adjusted to 10% (v/v) or 1.0% (v/v) in the assay system. Hemolytic activity was measured and calculated as previously described [50]. To measure the hemolytic activity of the prepore-locked recombinants, the reaction was carried out in the absence or presence of 10 mM DTT.

### Receptor binding inhibition assay

We used recombinants of DLY (rDLY) from *S. mitis* strain Nm-76 and other reference CDCs (rSm-hPAF, rILY, and rSLY [40,50],^50^) for the assay. The minimal protein concentration of each recombinant CDC showing complete hemolysis was adopted in this assay and adjusted with PBS to the final concentration of 1.4 nM for both rDLY and rSm-hPAF, 0.53 nM for rILY, and 0.56 nM for rSLY. To inhibit the binding between recombinant CDC and human CD59 on erythrocytes, we pre-incubated anti-human CD59 monoclonal antibody (YTH53.1; DS Pharma Biomedical Co., Ltd., Suita, Osaka, Japan) with human erythrocytes at a final concentration of 50 μg/mL for 15 min at 37°C and then washed them with PBS. Human erythrocytes pre-incubated with or without anti-human CD59 monoclonal antibody were added to the reaction mixture at a final concentration of 0.5% (v/v) and incubated at 37°C for 1 h. To inhibit the binding of recombinant CDC to membrane cholesterol, each recombinant CDC was pre-incubated (37°C, 15 min) with cholesterol at a final concentration of 1 μM in PBS before the assay. A sample containing 0.1% (v/v) ethanol was used as control for the cholesterol solvent. Each reaction mixture was incubated at 37°C for 1 h for the hemolysis assay, and the hemolytic activity was calculated as previously described [50].

### Cytotoxicity assay

The cytotoxicity of the culture supernatant of *S. mitis* strain Nm-76 and recombinant proteins to the human monocytic cell line THP-1 (RCB1189; Riken BioResource Research Center, Ibaragi, Japan) was assessed as previously described [47]. Briefly, THP-1 cells were cultured in RPMI1640 supplemented with 10% (v/v) heat-inactivated fetal bovine serum (FBS) and antibiotics (penicillin G and streptomycin), washed twice, and resuspended in RPMI1640 without both heat-inactivated FBS or antibiotics. Cells (5 × 10^4^ cells/well in 96-well plates) were mixed with culture supernatant (final concentration 10% (v/v)) or a series of dilutions of recombinant protein prepared in RPMI1640 and then incubated at 37°C for 1 h in a 5% CO_2_ atmosphere. The positive control (100% viable cells) was incubated with BHI instead of the culture supernatant or RPMI1640 instead of the recombinant protein solution. The negative control (0% viable cells) was prepared by adding 10 μL 1.0% (w/v) SDS solution to the cells. Thereafter, 10 μL of CCK-8 (Dojindo, Kumamoto, Japan) was added to each well and incubated at 37°C and 5% CO_2_ for 1 h. Color change was measured at 450 nm (reference wavelength at 600 nm) using an Infinite M200 microplate reader (TECAN, Männedorf, Zurich, Switzerland).

The CDC-dependent cytotoxicity of *S. mitis* strain Nm-76 and its mutant towards THP-1 cells was also evaluated applying propidium iodide (PI) staining under co-cultivation conditions using RPMI1640 (without phenol red) containing 10% (v/v) heat-inactivated FBS, 10% (v/v) BHI medium, and 25 mM HEPES (pH 7.4). THP-1 cells (1 × 10^5^ cells/well in 96-well plates) were co-cultivated with the tested bacteria (OD_600_ = 0.01 per well) and incubated at 37°C and 5% CO_2_ for 4 h. After adding PI at a final concentration of 1 µg/mL in the reaction mixture and incubating at 37°C and 5% CO_2_ for 15 min, the fluorescence intensity of PI was measured using a microplate reader (Infinite M200, TECAN) at excitation and fluorescence wavelengths of 530 nm and 620 nm, respectively. The PI fluorescence intensity measured in the well containing only cultured bacteria was subtracted from the PI fluorescence intensity measured in the co-cultivated well, and the net fluorescence intensity of THP-1 cells was shown as the percentage of PI fluorescence against the result of THP-1 cells without co-cultivation of bacteria as a control.

### Erythrocyte aggregation activity

A two-fold dilution series of prepore-locked recombinant CDCs (starting concentration of 10 nM) was prepared in PBS. We aliquoted 50 μL of each dilution into wells of a V-bottom 96-well plate and prepared a 2.0% (v/v) PBS suspension of human erythrocytes. Then, 50 μL of each suspension was added to the pre-dispensed recombinant protein solution and gently mixed. After incubation for 1 h at room temperature (20–25°C), erythrocyte aggregation was visually observed.

### Intercellular association assay

THP-1 cells showing green fluorescence were prepared by incubating 2 μg/mL calcein-AM (Dojindo) at 37°C and 5% CO_2_ for 15 min. After washing twice with PBS, the prepared fluorescent THP-1 cells were adjusted to 1.0 × 10^6^ cells/mL with RPMI1640. The fluorescent THP-1 suspension (500 μL) was mixed with an equal volume of prepore-locked recombinant CDC (55 nM) and incubated at 37°C and 5% CO_2_ for 1 h. The unbound recombinant proteins were removed by washing thrice with RPMI1640. To evaluate whether intercellular association depends on DD, we also performed the assay in the presence of AS against recombinant DD. In this experiment, fluorescent THP-1 cells with prepore-locked recombinant CDC were incubated with AS [49] or non-immune serum (NS) for 1 h, centrifuged (100 ×* g*, 3 min), and washed once with RPMI1640. The HepG2 human hepatoma cell line (RCB1648, Riken BioResource Research Center) was inoculated into a 48-well plate (0.8 × 10^5^ cells/300 μL/well) and incubated at 37°C overnight in a 5% CO_2_ atmosphere. After removing the culture supernatant and washing once with RPMI1640, the recombinant CDC-treated fluorescent THP-1 cells prepared as described above were added (0.3–0.6 × 10^5^ cells/300 μL/well) to each well and incubated at 37°C and 5% CO_2_ for 1 h. After removing the unbound THP-1 cells by washing thrice with 600 μL/well of Dulbecco’s modified Eagle’s medium (DMEM), the wells were observed using an IX71 microscope (Olympus, Tokyo, Japan). Next, 200 μL of 1.0% (w/v) SDS solution was added to each well to extract calcein from the cells. We transferred 100 μL of the lysate to a 96-well black plate and measured the fluorescence using a microplate reader (Infinite M200, TECAN) at an excitation and fluorescent wavelength of 477 nm and 535 nm, respectively.

### Enzyme-linked immunosorbent assay (ELISA)

The reactivity and specificity of AS raised against recombinant DD were evaluated using ELISA as previously described [49] with three recombinant proteins: rDLY(ss), ΔDD(ss), and recombinant DD as antigens.

### Statistics

Data were statistically analyzed using the R software for Mac OS X (R version 3.6.1, https://cran.r-project.org/bin/macosx/). The significance of differences between samples was evaluated using F-tests, followed by Student *t*-tests or Welch’s *t*-tests. Normality of the data was evaluated using the Kolmogorov–Smirnov test.

## Results

### Structural characteristics of discoidinolysin

We identified a 2,052 bp nucleotide sequence of the gene encoding DLY. Based on the deduced amino acid sequence of DLY, the N-terminal secretion signal was predicted using SignalP 5.0 (https://services.healthtech.dtu.dk/service.php?SignalP-5.0) shown as the underlined sequence in Figure 1a. Apart from the basic four-domain structure of CDCs, DLY possesses an additional N-terminal domain, designated as DD (Figure 1a,b, shown in magenta). This molecular structure is similar to those of Sm-hPAF and LLY [40,41]. A motif search using Pfam (http://pfam.xfam.org) revealed that the F5_F8_type_C domain (pfam00754) exists in DD. Interestingly, the gene encoding this domain is 21 bp longer than the part corresponding to the additional N-terminal domain of *llyA2* from *S. mitis* strain SK597 [29]. The connection of DD to domain 1 of DLY involves a longer flexible linker than those present in Sm-hPAF and LLY (Figure 1b). The amino acid sequence identity and similarity between DLY and the predicted product of *llyA2* was 93% (636/683) and 98% (674/683), respectively.

### Phylogenetic analysis of discoidinolysin

**Figure 2.**
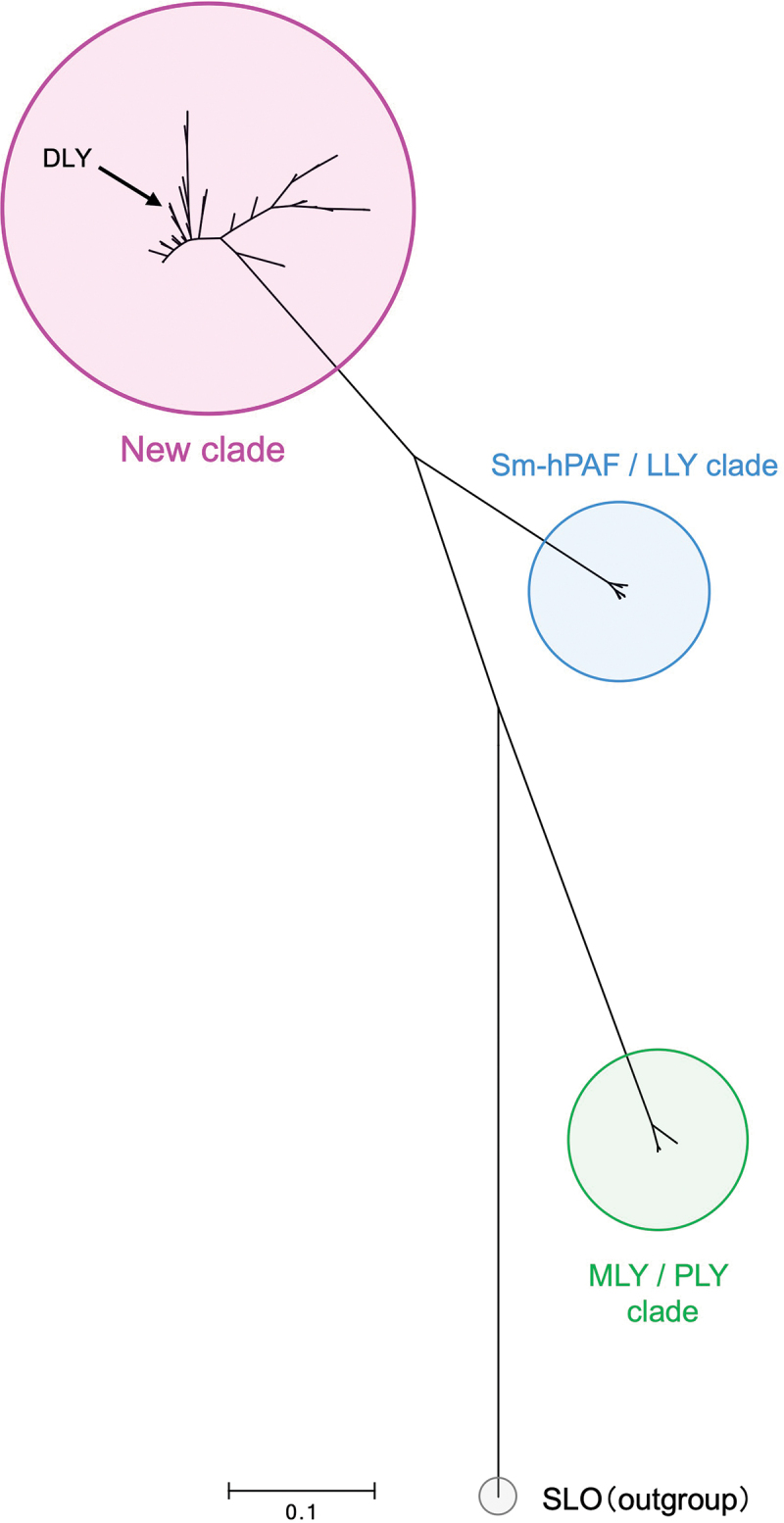
Phylogenetic analysis of the CDCs produced by S. mitis strains and related species among the Mitis group streptococci (MGS) based on nucleotide sequence. Streptolysin O (SLO) derived from S. pyogenes strain MGAS5005 was chosen as the outgroup in this analysis.

We performed BLAST analysis of the *dly* gene and detected its homolog in the *S. mitis* strains and some strains belonging to *S. pseudopneumoniae* (Table S2). Phylogenetic analysis indicated a new clade that included the DLY of strain Nm-76 and LLYA2 of strain SK597. This clade was mapped independently from the Sm-hPAF/LLY clade (Figure 2).

The distribution of genes encoding CDCs in *S. mitis* was also investigated using the *S. mitis* nucleotide BLAST database (https://www.ncbi.nlm.nih.gov/genome/?term=Streptococcus-mitis). Among the three types of CDC-encoding genes, the gene encoding DLY was most frequently found in *S. mitis* strains. Approximately 40% (56/143 strains) of the strains belonging to *S. mitis* possessed the *dly*/*llyA2* gene (Table 1). Thus, DLY has been implicated as the major CDC in *S. mitis* species.

**Table 1.** Distribution of the gene encoding each CDC among *S. mitis* strains.

Database	No. of gene-positive strains		
	DLY	Sm-hPAF/LLY	MLY
Complete genome (9 strains)	1	1	1
Draft genome (134 strains)	55	3	8

### DLY-dependent hemolytic and cytotoxic activities of S. mitis strain Nm-76

**Figure 3.**
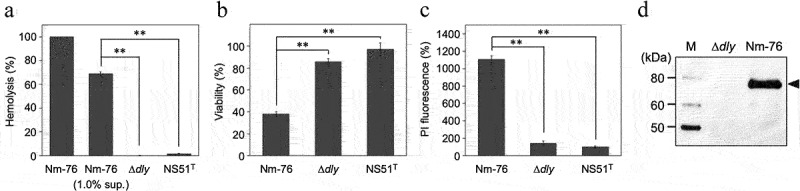
DLY-dependent human cell damage induced by bacterial culture supernatants and co-cultivation with bacterial cells. Hemolytic activity against human erythrocytes (a) and cytotoxicity against THP-1 (b) of supernatants prepared from overnight cultures of S. mitis strain Nm-76, its dly-deletion mutant (Δdly), and S. mitis type strain NS51T that does not possess the dly, were investigated and shown as hemolysis (%) and viability (%), respectively, relative to the control untreated with culture supernatant. (c) The DLY-dependent cytotoxicity of S. mitis strain Nm-76 against THP-1 was also evaluated by staining with PI under the co-cultivation conditions. The result is shown as PI fluorescence intensity (%) relative to the sample without co-cultivation with bacteria. PI fluorescence intensity derived from bacterial cells was subtracted from the fluorescence intensity measured in all sample cases. Samples were prepared in triplicates, and each was assayed twice. Representative results are shown as averages with standard deviations (SD). Significance of differences between S. mitis strain Nm-76 and Δdly mutant, or between Nm-76 and NS51T was evaluated using F-tests followed by Welch’s t-tests or Student t-tests (**p < 0.01). The production of DLY by Nm-76 and the Δdly mutant was evaluated using immunoblotting with antiserum (AS) against the DD as the primary antibody, horseradish peroxidase (HRP)-labeled anti-mouse IgG as the secondary antibody, and chemiluminescence detection reagent (Immobilon™ Western Chemiluminescent HRP Substrate, Millipore) (d).

We constructed the *dly*-deletion mutant (Δ*dly*) and investigated its phenotype to evaluate the contribution of DLY to *S. mitis* Nm-76 cytotoxicity. No hemolytic activity was observed in the culture supernatants of Δ*dly* (Figure 3a). In addition, the cytotoxicity of the culture supernatant towards THP-1 cells was also remarkably decreased after deleting *dly* (Figure 3b). We also observed significantly decreased THP-1 cell cytotoxicity of the Δ*dly* strain even under co-cultivation conditions (Figure 3c). The deficiency of DLY production in the Δ*dly* strain was confirmed using immunoblotting with AS against DD as the primary antibody (Figure 3d). These results indicate that DLY is solely responsible for β-hemolysis and cytotoxicity of *S. mitis* strain Nm-76 in human cells.

### Receptor recognition in hemolytic action by DLY

**Figure 4.**
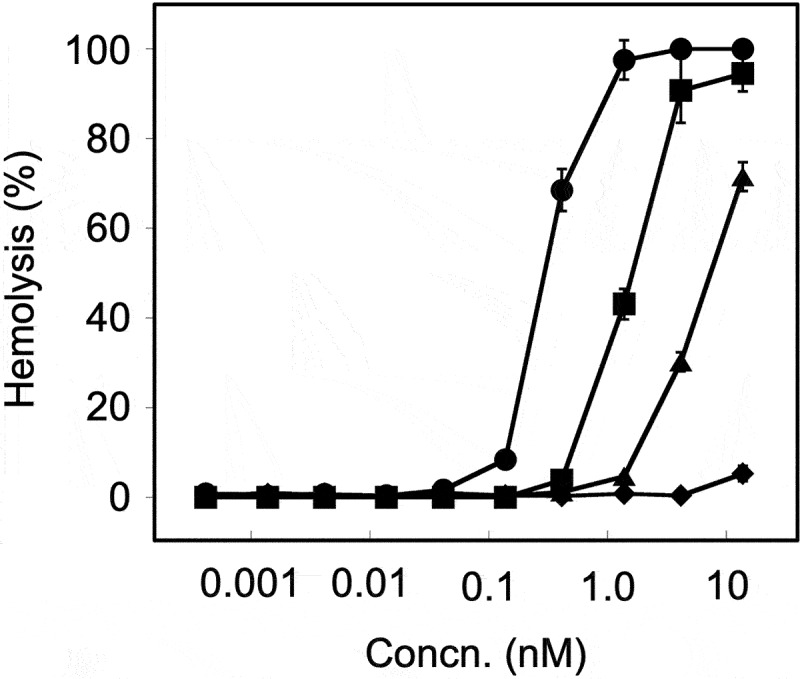
Species-dependent hemolytic activity of rDLY. Hemolysis dependent on the concentration of rDLY for erythrocytes of human (filled circles), horse (filled squares), rabbit (filled triangles), and sheep (filled diamonds) were investigated. Samples were prepared in triplicates and each was assayed twice. Representative results are shown as average hemolysis with standard deviation (SD).

We investigated the hemolytic activity of rDLY using erythrocytes from humans and animals (horses, rabbits, and sheep). As seen in Figure 4, rDLY showed the human erythrocyte-preferential and animal species-dependent hemolytic activity. The 50% hemolytic dose (HD_50_) against tested erythrocytes were: human erythrocytes, 0.3 nM; horse erythrocytes, 1.4 nM; rabbit erythrocytes, 7.1 nM; sheep erythrocytes, >13.8 nM (Figure 4).

**Figure 5.**
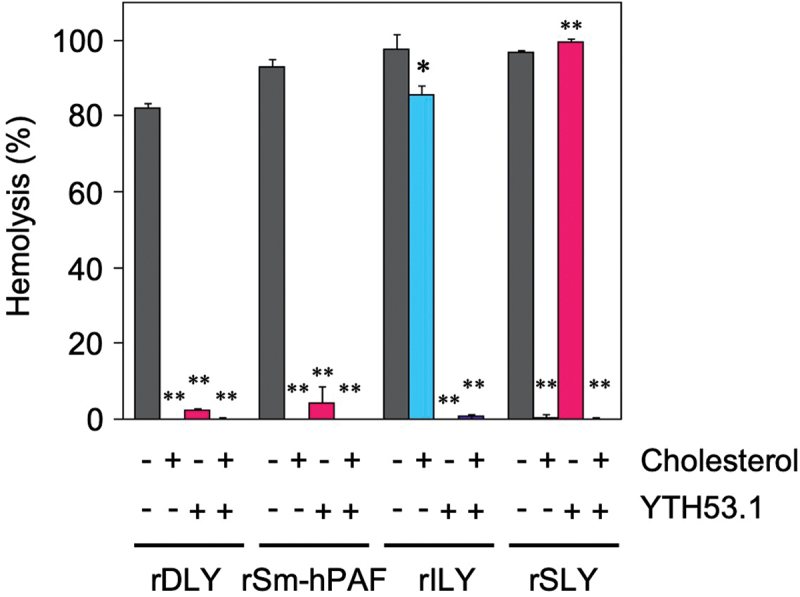
Mode of receptor recognition of DLY and other CDCs. Each recombinant CDC (rDLY, rSm-hPAF, rILY, and rSLY) was incubated with human erythrocytes in the presence or absence of cholesterol and/or human CD59 monoclonal antibody (YTH53.1). Triplicate samples were assayed at least twice each. Representative results are shown as hemolysis averages with standard deviations (SD). Significance of differences between hemolysis in the absence (dark-gray bar) and presence of inhibitor(s) for receptor binding of CDCs (cholesterol only, cyan bar), YTH53.1 only (magenta bar), and both (purple bar) was evaluated using F-tests followed by Welch’s t-tests or Student t-tests (**p < 0.01, *p < 0.05).

We performed a receptor binding inhibition assay of rDLY. In this assay, human erythrocytes were pre-incubated with anti-human CD59 monoclonal antibody (YTH53.1) to inhibit hemolysis by human CD59-recognizing CDCs such as ILY [52,53]. Then, a hemolysis assay was performed in the presence of cholesterol to inhibit the binding of CDCs to membrane cholesterol in human erythrocytes. An assay was also performed in the presence of both inhibitors. rDLY and other reference recombinant CDCs (rSm-hPAF, rILY, and rSLY) were incubated under the conditions described above, and hemolysis was measured and evaluated. As seen in Figure 5, rDLY showed a trend in the mode of receptor recognition like rSm-hPAF (belonging to Group III CDC), *that is,* its hemolytic activity was inhibited by the treatment of human erythrocytes with anti-human CD59 antibody or by the presence of cholesterol. However, the assay result of rDLY was different from both rILY (belonging to Group II CDC) and rSLY (belonging to Group I CDC). These results indicated that DLY is a dual-receptor recognizing CDC, like Sm-hPAF/LLY and vaginolysin (VLY) [50]. Therefore, DLY should be categorized as a Group III CDC.

### Membrane-binding and prepore-formation of DLY with intramolecular disulfide bond

**Figure 6.**
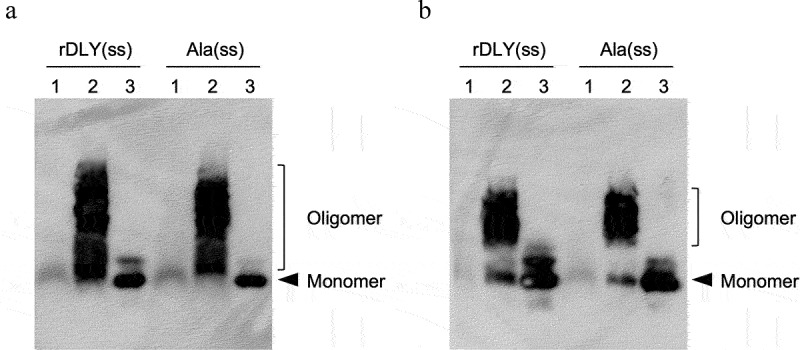
Activities of membrane-binding and prepore-formation of DLY-based recombinants possessing an intramolecular disulfide bond (rDLY(ss) and Ala(ss)). Each recombinant protein was incubated with human erythrocytes in the absence (a) or presence (b) of 10 mM DTT. Subsequently, the recombinant proteins bound on the erythrocytes were observed using immunoblotting with anti-DD antiserum after sodium dodecyl sulfate-agarose gel electrophoresis (SDS-AGE) of the reacted erythrocytes. Lanes: 1, without recombinant protein (erythrocytes only); 2, erythrocytes incubated with recombinant protein; 3, recombinant protein only.

In this study, three DLY mutants with an intramolecular disulfide bond causing conformational locking: rDLY (rDLY(ss)), and its two mutants (ΔDD(ss) and Ala(ss)) were used. We investigated the hemolytic activity of these mutants in human erythrocytes in the presence or absence of DTT. From the result of Figure S2, all the investigated mutants showed suppression of hemolysis in the absence of DTT, but significant hemolytic activity was observed in the presence of DTT. Subsequently, membrane-binding activity and prepore-formation activity were observed using immunoblotting after SDS-AGE separation. As shown in Figure 6, DLY mutants with an intramolecular disulfide bond (rDLY(ss) and Ala(ss)) formed an oligomeric structure, even in the absence of DTT (Figure 6a) and did not show obvious hemolytic activity (Figure S2). In addition, we could not investigate the membrane-binding and prepore-formation activities of ΔDD(ss) in this assay because the AS used as the primary antibody in this detection system was generated against DD as the antigen. However, we believe that ΔDD(ss) will show results like other mutants because ΔDD(ss) shares the same four domains as other mutants.

### N-terminal additional domain-dependent enhancement of aggregation of human erythrocytes

**Figure 7.**
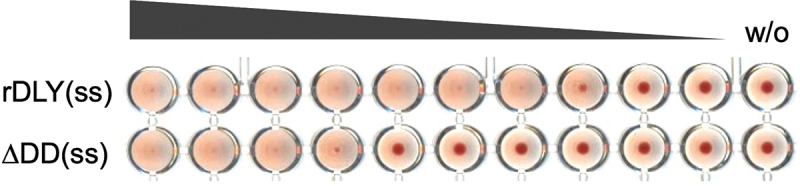
DD-dependent enhancement of human erythrocyte aggregation by rDLY. Each prepore-locked mutant of rDLY and its N-terminal domain-deletion mutant (rDLY(ss) and ΔDD(ss), respectively) was mixed with human erythrocytes and left static until erythrocyte aggregates formed. A representative result from several assays is shown.

To investigate the contribution of DD to the aggregation of human erythrocytes, two DLY recombinants with intramolecular disulfide bonds, rDLY(ss) and ΔDD(ss), were used (Figure S2). Since the erythrocyte aggregation activity of ΔDD(ss) was observed to be more than one order lower than that of rDLY(ss) (Figure 7) (the minimum concentration of ΔDD(ss) and rDLY(ss) required to aggregate human erythrocytes was 1.3 nM and 0.078 nM, respectively), it is obvious that erythrocyte aggregation is enhanced in the presence of DD.

### N-terminal additional domain-dependent intercellular association by DLY

**Figure 8.**
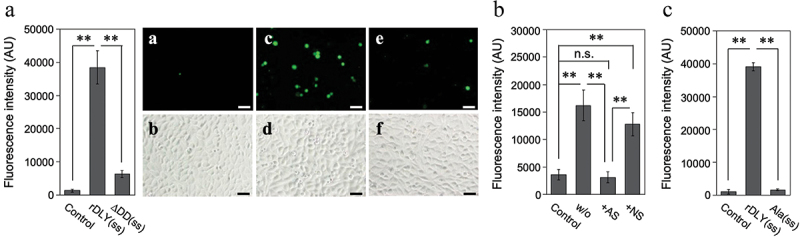
DD-dependent intercellular association mediated by DLY. (a) Dual-mode binding of DLY to human culture cell lines THP-1 and HepG2 was investigated using rDLY(ss) and ΔDD(ss). For visual comparison of the intercellular association between THP-1 and HepG2, photographs of typical fluorescent (a, c, e) and bright-field images (b, d, f) in this assay are shown for rDLY(ss) (c, d), ΔDD(ss) (e, f), and an assay of cells in the absence of recombinant protein (a, b). The scale bar denotes 50 μm. (b) The effects of incubating rDLY(ss)-treated THP-1 with antiserum (AS) against the DD or with non-immune serum (NS) on intercellular association due to dual-mode binding of rDLY(ss) were also investigated. (c) The effect of alanine substitutions at the three amino acids predicted to be responsible for the lectin activity on the intercellular association by rDLY(ss) were investigated. All bar graphs are shown as arbitrary fluorescence intensity measurements made with a microplate reader. Triplicate samples were assayed at least twice each. Representative results are shown as averages with standard deviations (SD). Significance of differences among the results shown was evaluated using F-tests followed by Welch’s t-tests or Student t-tests (**p < 0.01, n.s. indicates not significant).

The contribution of DD to intercellular associations among human cells was also investigated. For this assay, two human cell lines, HepG2 and THP-1, were used as the acceptor and indicator cells, respectively. The results showed that rDLY(ss) significantly induced intercellular association between THP-1 and HepG2 cells (Figure 8a). This association was diminished in the assay using ΔDD(ss) (Figure 8a). Moreover, this intercellular association induced by rDLY(ss) was significantly reduced by treatment with AS specific for DD (Figure 8b). This significant reduction was not observed in the NS group (Figure 8b). Alanine substitutions of three amino acid residues predicted to be important for lectin activity in the DD (Ala(ss)) almost completely inhibited this intercellular association (Figure 8c).

## Discussion

In the present study, we investigated the molecular characteristics of the novel CDC, discoidinolysin (DLY), produced by *S. mitis* strain Nm-76 (Figure 1). Previously reported CDCs produced by *S. mitis* strains include MLY [38], Sm-hPAF [40], and LLY [41]. In addition, Morales et al. reported the presence of genes encoding LLY-related proteins in several strains of *S. mitis* [29]. One of the genes, designated *llyA2* by the authors, displayed homology to the *dly* in *S. mitis* Nm-76 introduced in this study. However, production of the transcribed product from *llyA2*-positive strains has not yet been confirmed, and the molecular characteristics of the *llyA2* product remain unclear. Another *lly* related gene, *llyB*, has also been reported [29]. However, the product of *llyB* is not cytolysin but rather an adhesion molecule named mitilectin (MLC) [49]. The nomenclature of LLYA2 seems unsuitable for systematically distinguishing the *dly/llyA2* gene product from Sm-hPAF/LLY and LLYB/MLC. Therefore, in the present study, we designated the novel CDC found in *S. mitis* strain Nm-76 as DLY and performed molecular characterization of DLY.

Interestingly, the open reading frame of *dly* and its homolog was most frequently observed in *S. mitis* strains (Table 1). Therefore, we think that the most common CDC in *S. mitis* strains is DLY. In addition to the *S. mitis* strains, DLY was found in other related species deposited in the NCBI database (Table S2), as previously reported [29]. We investigated the actual expression of the gene and the participation of DLY in the virulence of *S. mitis* strain Nm-76 in human cells. The Nm-76 mutant devoid of the DLY-encoding gene did not express DLY (Figure 3d) and showed a non-hemolytic phenotype (Figure 3a). In addition, the DLY-deleted mutant was significantly less cytotoxic toward THP-1 human cells than wild-type Nm-76 (Figure 3b). Co-cultivation of THP-1 cells with *S. mitis* strain Nm-76 also induced significant cellular damage compared to the Δ*dly* mutant (Figure 3c). These results indicate that DLY is the factor responsible for β-hemolysis and cytotoxicity toward human cells infected with the strain Nm-76, suggesting that DLY is a key virulence factor in the β-hemolytic subgroup of *S. mitis*.

The molecular characteristics of DLY were determined using several recombinant proteins. Based on the mode of receptor recognition of domain 4 of DLY (Figure 5), DLY should be classified as a Group III CDC that recognizes both human CD59 and cholesterol as receptors for function [50]. This suggestion was also supported by the results of human-preferential and animal species-dependent hemolytic activity (Figure 4) and by the amino acid alignment of domain 4 (Figure S3). Because the previously reported five-domain CDCs (Sm-hPAF and LLY) are also categorized in Group III [50], the atypical five-domain CDCs produced from MGS have a common mode of receptor recognition involving the dual recognition of human CD59 and cholesterol. Moreover, since either masking of human CD59 on target cells or the binding site for cholesterol of Group III CDCs drastically reduced their hemolytic activity (Figure 5), both receptors are necessary for the pore-forming action of Group III CDCs. Reportedly, the cytotoxicity of typical CDCs categorized in Group I that recognizes only cholesterol as their receptor is inhibited in the presence of cholesterol *in vivo*, such as in the blood. However, Group II CDC ILY, which recognizes human CD59 as its receptor, can maintain its cytotoxic activity in a cholesterol-rich environment *in vivo*. Taken together, Group III CDCs seem to maintain the prepore structure after binding to human CD59 *in vivo* and may cause cell damage when the concentration of free cholesterol in the blood/lymph liquid is lowered. This possibility is interesting when considering the function of Group III CDCs, as discussed below.

Although DD hardly contributed to hemolytic activity toward human erythrocytes and cytotoxicity toward THP-1 human acute monocytic leukemia cells (Figure S4), DD enhanced the aggregation of human erythrocytes (Figure 7) and intercellular association of human cell lines (Figure 8). Because no significant difference was found in the results of the titration curves in the hemolysis assay between rDLY and ΔDD (Figure S4a), the affinity of DLY for the cell membrane *via* domain 4 seems to be higher than that *via* DD. Thus, the DLY monomer binds to the cell membrane primarily *via* domain 4 and subsequently oligomerizes on the membrane to form a prepore structure. Although there is no high-resolution structural data of the membrane pore of DLY, it is suggested that several dozens or so DD would be arranged in a circle on the target cell surface by the formation of prepores or mature pores (Figure 6). Thus, the formation of prepores or mature pores may contribute to the avidity by clustered DD in the pore structure and induce intercellular association. This suggestion is supported by the observation that AS against DD (Figure S5) interfered with intercellular association by DLY (Figure 8b). Moreover, this intercellular association is presumably attributed to the lectin activity of the DD, because the alanine-substituted mutations of DLY significantly decreased the intercellular association (Figure 8c). Taken together, these results suggest that DLY functions not only as a hemolytic/cytotoxic factor but also as an association molecule in human cells *via* their N-terminal DD in addition to the C-terminal domain 4. Therefore, if *S. mitis* strains producing DLY and their homologs translocate into the blood and proliferate – in addition to DLY-dependent hemolysis and functional failures in the cells, such as neutrophils, macrophages, and vascular endothelial cells – the aggregation of blood cells and/or association between blood cells and vascular endothelial cells are suggested to occur *in vivo*. This might explain or give a working hypothesis for the mechanism of ectopic infection that induces and enhances inflammation and pathogenicity of *S. mitis*. Further investigations on the mode of action of DLY and other Group III CDCs produced from the β-hemolytic subgroup of *S. mitis* both *in vitro* and *in vivo* are needed to elucidate the detailed mechanism of pathogenicity of the β-hemolytic subgroup of *S. mitis* strains.

Recently, the CDC-dependent potential pathogenicity of the *S. mitis* strain Nm-65 isolated from a patient with Kawasaki disease has been reported [47]. Nm-65 produced two different CDCs (MLY and Sm-hPAF/LLY) that contributed to hemolytic activity and cytotoxicity. In the present study, we revealed the molecular characteristics of DLY produced by *S. mitis* strain Nm-76 that is phylogenetically different from other *S. mitis* producing CDCs: MLY and Sm-hPAF/LLY. Interestingly, as mentioned above, MLC was also reported to be an adhesion molecule with tandem F5_F8_type_C domains supporting bacteria-host interactions [49]. The gene encoding MLC frequently co-exists with the gene encoding CDCs, including DLY, in the genomes of *S. mitis* strains [29]. Therefore, the synergistic pathogenicity of MLC and CDCs should be investigated to understand the interaction between *S. mitis* and human cells. Interestingly, strain Nm-76 was isolated from a patient with Kawasaki disease. This is suspected to be an infectious disease; however, its etiological agent remains unclear. The results shown in this study suggest some contribution of a β-hemolytic CDC-producing subgroup of *S. mitis* strains to Kawasaki disease. Furthermore, the significant diversity of CDCs in the strains of *S. mitis* and related species suggests that MGS that habitually reside in humans may serve as a genetic reservoir for genes encoding virulence factors, such as CDCs and related molecules. Re-evaluation of the potential pathogenicity of human habitual *S. mitis* and related bacteria will be important and beneficial for basic studies on *S. mitis* and other human opportunistic streptococci and will also provide useful clinical information concerning infectious diseases.
